# RNA-Seq Analysis of *Mycobacterium avium* Non-Coding Transcriptome

**DOI:** 10.1371/journal.pone.0074209

**Published:** 2013-09-16

**Authors:** Dmitriy Ignatov, Sofia Malakho, Konstantin Majorov, Timofey Skvortsov, Alexander Apt, Tatyana Azhikina

**Affiliations:** 1 Shemyakin and Ovchinnikov Institute of Bioorganic Chemistry, Moscow, Russia; 2 Center of Innovations and Technologies “Biologically Active Compounds and their Applications”, Moscow, Russia; 3 Faculty of Biology, Lomonosov Moscow State University, Moscow, Russia; 4 Central Institute for Tuberculosis, Moscow, Russia; St. Petersburg Pasteur Institute, Russian Federation

## Abstract

Deep sequencing was implemented to study the transcriptional landscape of *Mycobacterium avium*. High-resolution transcriptome analysis identified the transcription start points for 652 genes. One third of these genes represented leaderless transcripts, whereas the rest of the transcripts had 5′ UTRs with the mean length of 83 nt. In addition, the 5′ UTRs of 6 genes contained SAM-IV and Ykok types of riboswitches. 87 antisense RNAs and 10 intergenic small RNAs were mapped. 6 intergenic small RNAs, including 4.5S RNA and rnpB, were transcribed at extremely high levels. Although several intergenic sRNAs are conserved in *M. avium* and *M. tuberculosis*, both of these species have unique intergenic sRNAs. Moreover, we demonstrated that even conserved small RNAs are regulated differently in these species. Different sets of intergenic sRNAs may underlie differences in physiology between conditionally pathogenic *M. avium* and highly specialized pathogen *M. tuberculosis*.

## Introduction

Infections caused by mycobacteria, other than *Mycobacterium tuberculosis* and *M. leprae*, pose a serious medical problem. At least 60 species of environmental mycobacteria are known to cause opportunistic infections in humans [1], and *M. avium* is of special interest because of its environmental diversity and ability to infect birds, animals and immune compromised humans. According to the current taxonomy, *M. avium* includes 4 subspecies, *M. avium avium* (MAA), *M. avium hominissuis* (MAH), *M. avium silvaticum* (MAS), and *M. avium paratuberculosis* (MAP) [2], [3]. MAA and MAS are specific avian pathogens causing a tuberculosis-like disease in birds [4], whereas MAP is a well-known pathogen causing Johne’s disease, i.e., chronic enteritis of ruminants [5]. MAH causes disseminated infections in patients with severely compromised T cell immunity, and pulmonary infections in children and aged individuals [6], [7], [8], [9]. After entering the host, *M. avium* is engulfed by macrophages and resides within phagosomes, demonstrating capacity to block phagosome-lysosome fusion and prevent acidification of the internal phagosomal compartment, similarly to *M. tuberculosis* [10]. Although in humans major features of acquired immunity against *M. tuberculosis* and *M. avium* are similar, *M. tuberculosis* is much more aggressive and virulent, whereas *M. avium* affects only immune compromised individuals [11].

In the past decade, studies have revealed abundant non-coding transcriptomes in bacteria. Non-coding transcripts, including 5′UTRs, antisense transcripts, and intergenic small RNAs, primarily play roles in the regulation of gene expression. Pathogenic bacteria encounter diverse environmental conditions and therefore require fast regulatory circuits to survive. Together with regulatory proteins and 2-component systems, regulatory RNAs allow pathogens to adjust their metabolism and express factors that are necessary for subverting host defense [12]. 5′ UTRs in bacteria are of varying lengths reaching several hundred nucleotides. They can alter their conformation in response to changes in temperature, pH, or metabolite concentrations. Changes in conformation subsequently regulate the expression of downstream genes by transcription termination/antitermination or blocking/unblocking of ribosome-binding sites [13]. Intergenic sRNAs, which are relatively short transcripts (∼50–300 nucleotides), act on distantly encoded targets. Most of them regulate mRNAs through short, imperfect base-pairing interactions, whereas others, e.g., *Escherichia coli* 6S and CsrB RNAs, modify protein activities [14]. Antisense transcription may be generated by either bona fide antisense RNAs (asRNAs), which do not encode proteins, or by overlapping parts of mRNAs [15]. It has been reported that antisense transcription may regulate the transcription and translation of coding transcripts. An attractive model for the activity of antisense transcripts is the digestion of antisense-coding RNA duplexes by RNase III [23].

Investigations of mycobacterial non-coding transcriptomes have focused mainly on *M. tuberculosis*. The first mycobacterial small RNAs were mapped in *M. tuberculosis* by cloning and sequencing a fraction of short RNAs [16], as well as in *M. bovis* BCG by using a combination of computer prediction and cloning strategies [17]. Recently, bacterial RNA-seq has emerged as an accurate tool for studying bacterial transcriptomes [21]. For instance, Pellin et al. performed high-throughput sequencing of sRNA fractions of *M. tuberculosis* and identified ∼2,000 sRNA candidates by combining information regarding the read coverage with conservation analysis of intergenic regions [18]. A subsequent microarray analysis further confirmed the expression of 258 of these sRNA candidates, including 22 intergenic sRNAs and 152 antisense RNAs [19].

Arnvig et al. utilized RNA-seq for *M. tuberculosis* whole-transcriptome profiling at the exponential and stationary phases of growth and demonstrated abundant anti-sense transcription generated by overlapping 3′ UTRs and distinct asRNAs [20]. Overall, in *M. tuberculosis*, more than 20 intergenic sRNAs were identified and mapped [16], [17], [20], [21], and for a few of these sRNAs, implications in pathogenesis have been suggested. The levels of MTS194, MTS479, and MTS2822 were found to be increased in response to H_2_O_2_ mimicking oxidative stress inside host macrophages. Accumulation of MTS997, MTS1338, and MTS2823 at high levels was demonstrated during the transition from exponential to stationary phases of growth and along the course of infection [17], [21]. There is little doubt that intergenic sRNAs play an important role in mycobacterial physiology; however, the mechanism of their action remains unknown. Thus, *Mycobacteria* lack the Hfq protein facilitating interactions between intergenic sRNAs and their targets in gram-negative microorganisms [22].

Until recently, no data were published on non-coding transcriptome of *M. avium*, prompting us to fill this gap in our knowledge. By cloning and sequencing a fraction of short RNAs, we identified several trans-encoded sRNAs in *M. avium* subsp. *avium* TMC724 (ATCC25291) [23]. In the present study, we utilized a more powerful RNA-seq approach to profile the transcriptome of *M. avium* TMC724 in the mid-logarithmic growth phase. The single-nucleotide resolution data enabled us to identify multiple non-coding transcripts and exact transcript boundaries. In addition, 2 intergenic sRNAs with the highest *in vitro* expression levels were evaluated in the mouse infection model by means of qRT-PCR, and demonstrated profound expression differences in the lung tissue of genetically susceptible and resistant mice.

## Results and Discussion

### The total amount of intergenic transcription exceeds the transcription of protein-coding sequences in logarithmically grown *M. avium*

We used Illumina sequencing to study the transcriptome of *M. avium* TMC724. RNA was isolated from bacteria grown to the mid-exponential growth phase. 16S and 23S rRNA were removed with MicrobExpress kit (Life Technologies). To prepare cDNA for sequencing we utilized the Illumina RNA ligation protocol, which allows not only investigation of bacterial transcriptome in an unbiased manner but also preservation of information regarding the transcription directionality [24]. This protocol have shown a high level of technical reproducibility [25]. At present, complete sequencing of *M. avium* TMC724 genome is not finished yet. GenBank contains 258 contigs of the genome that were produced in a whole genome shotgun sequencing project under the accession number ACFI00000000. Although this genome is automatically annotated, we mapped our RNA-seq reads to the genome of another *M. avium* strain MAH104 (GenBank accession no. CP000479.1), since the genome sequence of the latter was completed and annotated. Our sequencing approach provided 42.2 million reads, 65–76 nucleotides (nt) in length. The mapping statistics is shown in Table 1. Totally, 14 million reads did not map to the *M. avium* MAH104 genome, whereas 18.8 million reads mapped to a single rRNA operon. Such a large number of unmapped reads (33%) may be explained by technical artifacts of sequencing.

**Table 1:** Mapping statistics.

	RNA-seq results, mln of reads	% of total	% of CDS	% of CDS (*M. tuberculosis*)
**total mapped reads**	28,2			
**reads mapped to rRNA operon**	18,8	67		
**reads mapped to CDS**	3,6	13		
**reads antisense to CDS**	0,2	0,7	5,4	16,1
**reads mapped to IGR**	4,4	16	123,1	23,4

Number of reads mapped to rRNA operon, CDS in sense and antisense orientation, intergenic regions (IGR) are shown in the first column. Percentages of reads mapped to these loci relative to all mapped reads are shown in the second column. In the third column percentages of reads mapped to CDS in antisense orientation and IGR relative to the number of reads mapped to CDS are represented. In the fourth column the same values for *M. tuberculosis* are shown [20].

In addition, while 3.6 million reads mapped to coding sequences (CDS) in the sense orientation, only 0.2 million reads mapped to CDS in the antisense orientation (5.4% of that mapping in sense). In contrast, 4.4 million reads, mapping to intergenic loci, constituted 123% of that mapping to CDS in sense (Table 1). Below, we show that such an enormous level of intergenic transcription is caused by several highly expressed small RNAs. Arnvig et al. have shown that in log-phase grown *M. tuberculosis*, reads mapping antisense to CDS constituted 16.1% of those mapping to CDS in the sense orientation, whereas those mapping to IGR constituted 23.4% of those mapping to CDS in the sense orientation [20]. Discrepancy in the amount of reads mapping antisense to CDS or to intergenic loci between our studies may be explained by different techniques of sample preparation for sequencing or by true differences in physiology between these microorganisms.

For rapid and convenient visualization of RNA-seq data, we produced the so-called transcriptional profile. For each nucleotide of the genome, we counted the number of reads overlapping the nucleotide. Such transcriptional profiles were made for forward and reverse strands of the genome and visualized in the Artemis genome browser [26].

#### The genome of *M. avium hominissuis* strain 104 possesses 25 large sequence polymorphisms, absent in *M. avium avium* TMC724

The major source of *M. avium* strain diversity is known to be their genomic heterogeneity, mainly large sequence polymorphisms (LSPs), which are genomic regions present in some strains but not others [27]. To identify *M. avium* 104 genomic loci that are potentially absent in *M. avium* TMC724, we aligned 258 contigs of the *M. avium* TMC724 genome with the complete sequence of the *M. avium* 104 genome. As a result, 25 such loci with lengths of more than 500 nt were found (Table 2). We observed that almost no reads mapped to these loci and that each locus was located in the gaps of *M. avium* TMC724 contigs. On the basis of these findings, we consider these loci to be LSPs specific to *M. avium* 104, but not *M. avium* TMC724. Totally, these 25 loci make up 750 kb of genetic material and contain 808 genes. Because we mapped the reads on genome of *M. avium* 104, 120 kb of the genetic material containing 165 genes that were present in *M. avium* TMC724, but not *M. avium* 104, were excluded from the examination. The examination of genes present in *M. avium* 104-specific LSPs suggests that at least some of them were acquired by horizontal transfer. LSPs 2, 5, 6, 9, and 22 are located adjacent to tRNA genes, which are often associated with phages [28]. LSPs 2, 5, 6, and 24 contain genes coding for site-specific recombinases/phage integrases, located just at the borders of these LSPs: MAV_0253, MAV_0698, MAV_0779, and MAV_3790, respectively. Interestingly, LSPs 2, 6, 9, and 12 also contain genes coding for viral proteins: LSP 2, MAV_0287 coding for gp6; LSP 6, MAV_0786, MAV_0787, and MAV_0792 coding for gp53, gp50, and gp34, respectively; LSP 9, MAV_1486, MAV_1501, and MAV_1504 coding for gp54, gp13, and gp2, respectively; and LSP 12, MAV_2256 coding for gp108. These data reveal that LSPs 2, 5, 6, 9, 12, and 24 are highly likely to have been acquired by *M. avium* subsp. *hominissuis* after its divergence from *M. avium* subsp. *avium*, as a result of horizontal genetic transfer. Interestingly, in contrast to pathogenic *M. avium* TMC724, conditionally pathogenic *M. avium* 104 contains 2 additional clusters of *mce* genes, MAV_2532-2537 and MAV_5047-5051 located in LSPs 14 and 22, respectively. The *mce* operons have been implicated in the pathogenesis of mycobacteria, and their products were suggested to be transporter systems [29], [30], [31], [32].

**Table 2:** Regions of MAH104 genome predicted to be absent in MAA TMC724.

Locus	start	end	tRNA	genes	transposon functions genes
LSP 1	28227	30559	−	MAV0029, MAV0030	−
LSP 2	254272	294337	+	MAV0253-MAV0298	+
LSP 3	321348	323021	−	MAV0330	−
LSP 4	462328	493802	−	MAV0472-MAV0507	+
LSP 5	665471	675801	+	MAV0684-MAV0698	+
LSP 6	746456	794401	+	MAV0779-MAV0841	+
LSP 7	1418088	1419076	−	MAV1449	−
LSP 8	1420005	1441044	−	MAV1452-MAV1476	+
LSP 9	1443844	1463419	+	MAV1481-MAV1505	+
LSP 10	1795159	1990411	−	MAV1799-MAV2006	+
LSP 11	2220300	2241547	−	MAV2217-MAV2234	+
LSP 12	2257518	2271617	−	MAV2248-MAV2266	+
LSP 13	2462680	2466685	−	MAV2438-MAV2441	−
LSP 14	2548414	2723551	−	MAV2515-MAV2687	+
LSP 15	2725237	2731207	−	MAV2691-MAV2698	+
LSP 16	2815770	2821500	−	MAV2788-MAV2793	−
LSP 17	3009919	3035725	−	MAV2973-MAV2998	+
LSP 18	3163322	3169333	−	MAV3097-MAV3102	−
LSP 19	3393282	3399655	−	MAV3251-MAV3257	+
LSP 20	3524728	3527412	−	MAV3379-MAV3381	−
LSP 21	3589447	3592532	−	MAV3443-MAV3446	−
LSP 22	3668848	3675750	+	MAV3531-MAV3536	+
LSP 23	3773828	3776445	−	MAV3646-MAV3648	−
LSP 24	3917475	3939505	−	MAV3790-MAV3809	+
LSP 25	5181203	5270251	−	MAV5030-MAV5116	+

Coordinates of starts and ends of the predicted Large Sequence Polymorphisms (LSPs) are shown according to genome of MAH104. Presence or absence of tRNA genes in MAH104 genome adjacent to borders of LSPs is shown. MAH104 genes located inside each of the LSPs and presence or absence of transposon functions genes among them is shown.

### 76% of *M. avium* genes are transcribed at a considerable level

RPKM, a measure of relative gene expression [33], normalizes the number of reads mapping to a gene to both the feature lengths and the total number of reads in an experiment. For the total number of reads in the experiment, we chose all mapped reads, except those mapping to the rRNA operon. We excluded these reads because of rRNA depletion, which may skew RPKM values. On the other hand, we included reads representing the non-coding transcriptome, because it includes important un-annotated features. Interestingly, the distribution of RPKM values for all genes was continuous; that is, despite significant variability in RPKM values, almost all genes were transcribed, and there were only 71 genes with a RPKM value of 0. Among these, 53 encode transposases and 6 (MAV_0763, MAV_2077, MAV_2417, MAV_2855, MAV_4320, and MAV_4779) contain short ORFs encoding putative proteins. However, these proteins have no homologs based on a psi-blast search; therefore, these 6 non-transcribed genes are probably the results of misannotation. The RPKM values for all annotated CDSs present in the MAA TMC724 genome are given in Table S1.

Transcription initiation is a stochastic event; even repressed genes are transcribed in a certain population of cells. Nevertheless, there is a possibility that a low level of transcription detected for a subpopulation of genes results from technical artifacts of sample preparation or the mapping of reads. To account for possible technical noise, we defined a gene to be transcribed when its RPKM value is more than 7. In the absence of additional information (e.g., information regarding expressed proteins [25]), the choice of such a threshold is somewhat arbitrary. A RPKM value of 7 was chosen because genes that have lower RPKM tend to have higher transcription from the antisense strand than that from the sense strand. Accordingly, we consider that 3,408 of 4,501 genes were transcribed and represent 76% of the genes under consideration. Similar results were obtained for *M. tuberculosis*; 78% of annotated genes were transcribed [20].

**Figure 1:**
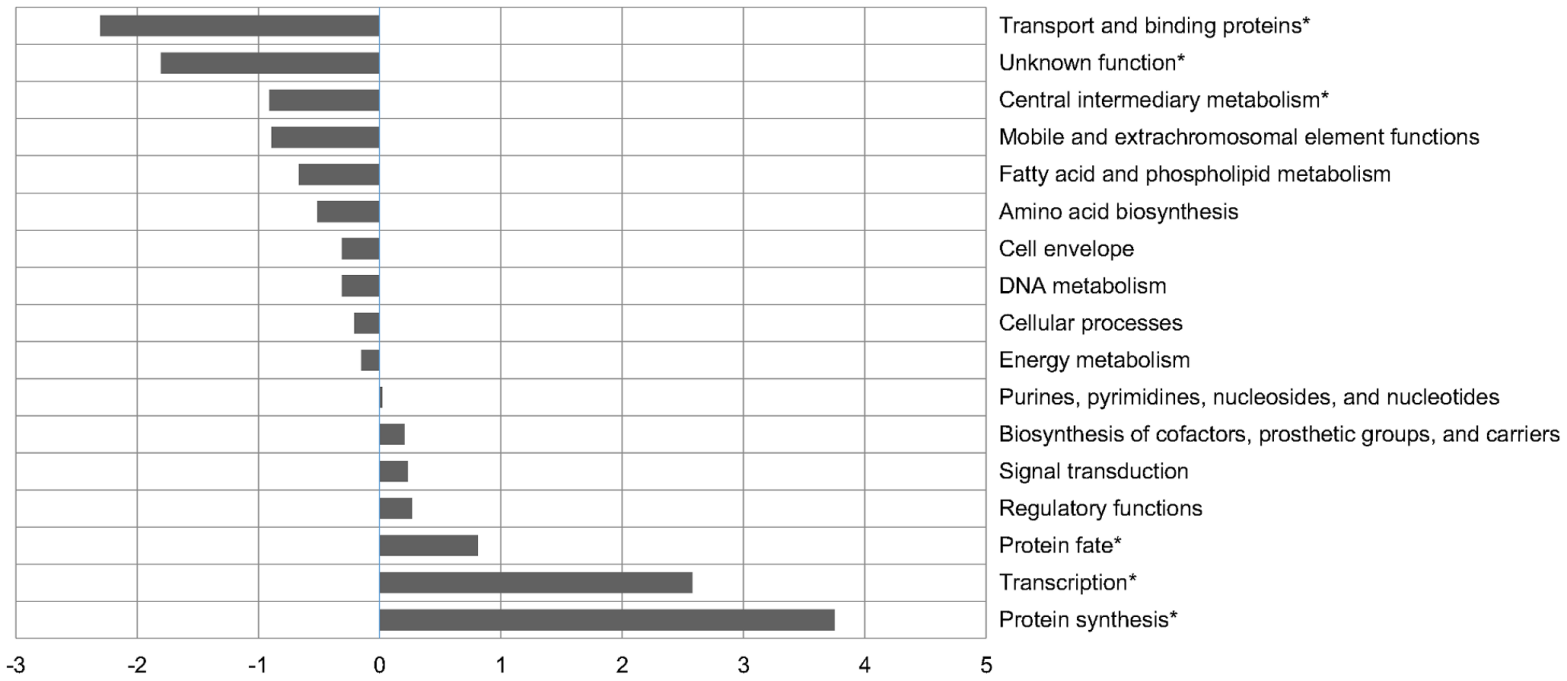
Functional classes of genes with the highest transcription. Bars show the levels of over- or underrepresentation of each functional category among 450 genes with the highest expression. Functional categories significantly over- or underrepresented in this list of genes are indicated with asterisks.

To study the relative transcription of genes representing different functional categories, we chose 10% of genes with the highest RPKM values and studied which functional classes were overrepresented among these gene groups (Figure 1).

The functional classes for *M. avium* 104 were retrieved from www.TIGR.org. Their over- or underrepresentation among the 450 genes with the highest RPKM values was confirmed by a binomial test with correction for multiple comparisons. According to the statistical criterion, genes encoding products participating in the protein synthesis, transcription, and protein fate were significantly overrepresented, whereas genes coding for transport and binding proteins, as well as proteins with unknown functions and proteins participating in central intermediary metabolism, were significantly underrepresented. Overall, such a picture is typical for bacteria grown in the logarithmic phase, since frequent cell divisions require the machinery for transcription and synthesis of new proteins. In contrast, in nutrient-rich media, the expression of genes encoding transport and binding proteins and enzymes involving in central intermediary metabolism is not necessary.

### One third of the revealed transcriptional start points correspond to leaderless transcripts

**Figure 2:**
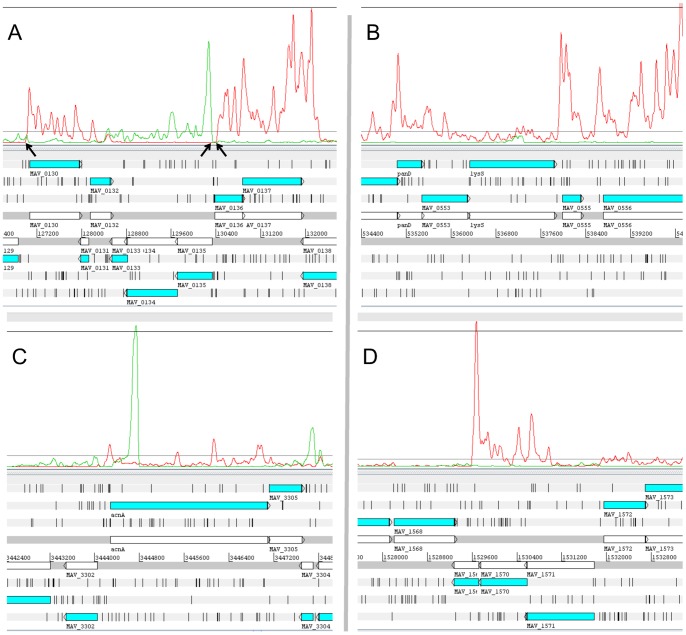
Transcriptional start points and cis-ncRNAs. Genomic loci and transcriptional profiles were visualized in the Artemis genome browser. Transcription from the forward or reverse strand of the genome is shown in red or green lines, respectively. Genes are expressed as blue boxes, with arrowheads pointing toward the right or left for genes encoded by the forward or reverse strand, respectively. (A) Genome locus comprising genes MAV_0130–MAV_0137. Identified TSPs for genes MAV_0130, MAV_0135, and MAV_0136 are indicated by arrows. (B) asMAV_0554. This antisense RNA to the gene lysS is transcribed at a level lower than lysS. (C) asMAV_3303. The level of transcription of this antisense RNA is much higher than that of acnA. (D) asMAV_1569–1571. This antisense RNA is transcribed at a relatively high level and spans 3 genes. Transcription of the genes antisense to asMAV_1569–1571 is negligible.

Visual inspection of the Artemis transcriptional profile led to the identification of abrupt increases in transcription levels, frequently located near the 5′ ends of annotated genes (Figure 2A). The positions of these spikes were considered as transcriptional start points (TSP). To map these points, we used a computer algorithm that accounted for the differences in transcription levels upstream and downstream of the spikes, the abruptness of increases, and the distance to the nearest upstream spike. All the mapped putative TSPs were manually verified. The spikes located upstream of the 5′ ends of annotated genes were considered to be their respective TSPs. In total, putative TSPs for 844 genes were mapped. Our method does not guarantee that all revealed TSPs are primary and not the products of degradation. To confirm their primary nature, we searched for –10 consensus sequences upstream of these TSPs using MEME software [34]. Such a consensus sequence was found to be located 5–8 nt upstream of TSPs of 652 genes and represents a hexamer, TA[GCA][GC][CG]T, with the first, second, and sixth nucleotide positions being the most conserved. Although many genes of mycobacteria are operated by sigma-factors alternative to SigA and therefore have another –10 consensus sequence, we discarded putative TSPs for genes that do not have properly positioned –10 sequences. To validate our method, 5 of 652 remained TSPs (MAV_1369, MAV_3090, MAV_4124, MAV_0525, and MAV_1669) were randomly selected and confirmed by RLM-RACE. TSPs revealed by inspecting the profile mapped ±3 nt from those revealed by RLM-RACE. A list of TSPs for 652 genes is summarized in Table S2. Interestingly, 33% of revealed TSPs mapped ±3 nt from the start codons of their respective genes; therefore, we consider that these genes are leaderless. Leaderless genes are widespread in *Actinobacteria*, and constitute approximately 20% of genes in this phylum [35]. Leaderless mRNAs may have an important role in stress adaptation in bacteria [36], and their abundance in *M. avium* requires further studies.

We have shown that M. avium contains even higher proportion of leaderless genes, than the average for *Actinobacteria*. The 5′ UTRs of other mRNAs varied from 3 to 728 nt in length, with a mean value of 83 nt. These 5′ UTRs may contain sequences that play a role in the regulation of gene expression.

### Ykok and SAM-IV riboswitches in *M. avium*

Visual inspection of long 5′ UTRs further identified sequences that possibly code for riboswitches, 4 of which matched to known riboswitch structures in the RFAM database (Table 3) [37]. The SAM-IV riboswitch was found upstream of MAV_4315. SAM-IV riboswitches specifically bind *S*-adenosylmethionine, a cofactor used in many methylation reactions [38], whereas MAV_4315 encodes *O*-acetylhomoserine sulfhydrylase, an enzyme implicated in cysteine and methionine metabolism.

**Table 3:** Predicted riboswitches.

Start	End	Gene	Product	Type
4445976	4446194	MAV_4315	O-acetylhomoserine sulfhydrylase	SAM-IV
2947367	2947501	MAV_2915	PE family protein	Ykok leader
3338199	3338383	MAV_3236	hypothetical protein	Ykok leader
5347431	5347601	MAV_5197	manganese/iron transporter	Ykok leader
2941785	2942378	MAV_2910	PPE family protein	putative riboswitch

Coordinates of the riboswitches are provided according to MAH104 genome. Genes downstream of the riboswitches and regulated by them, products of these genes and types of riboswitches according to RFAM database are demonstrated.

Ykok leader sequences were identified upstream of 3 following genes: MAV_2915, encoding a proline-glutamate (PE) protein; MAV_3236, a hypothetical protein; and MAV_5197, a manganese/iron transporter. The Ykok leader is a Mg^2+^-sensing structure that controls the expression of magnesium ion transport proteins in bacteria [39]. The most intriguing finding is the identification of a Mg^2+^-sensing riboswitch upstream of the putative operon encompassing MAV_2912–MAV_2915 genes, which encode a PE protein (MAV_2915), 2 proline-proline-glutamate (PPE) proteins (MAV_2913 and MAV_2914), and a conserved hypothetical protein (MAV_2912). The close proximity between these genes suggests that they constitute a single operon. PE/PPE proteins, possessing conserved proline-glutamate or proline-proline-glutamate motifs at their N terminus, are secreted or localized to the cell surface. While the function of these proteins is not distinctly known, several of them are thought to be virulence factors [40]. The presence of the Ykok leader reveals that this operon is activated upon Mg^2+^ starvation, which could be observed inside phagosomes of macrophages. In *M. tuberculosis*, the Ykok leader has been found upstream of a putative operon encoding 4 PE/PPE genes, a conserved hypothetical protein, and a predicted magnesium transporter, MgtC [21]. These data indicate that the expression of PE/PPE proteins may be regulated by the presence of Mg^2+^.

A putative riboswitch, which regulates the operon MAV_2907–MAV_2910, was revealed in the 5′ UTR of MAV_2910. Although its sequence did not match any known riboswitches in the RFAM database, the appropriate length of the 5′ UTR, as well as the high level of its transcription and decrease in transcription level downstream of it, suggest a bona fide riboswitch. This operon also encodes 2 PPE proteins and 2 proteins with unknown functions.

### Multiple non-coding RNAs revealed and mapped in *M. avium*

According to the mapping results, non-coding transcriptome constitutes a significant part of mycobacterial RNA. Sequencing allowed us to reveal dozens of putative antisense RNAs, i.e., transcripts with TSPs located antisense to annotated features (Table S3) and several intergenic small RNAs.

#### Antisense RNAs

To search for TSPs of putative antisense RNAs, we implemented the same algorithm used for searching TSPs of annotated genes. We manually verified the identified candidate TSPs but did not filter them by the presence of downstream start-codons or upstream –10 sequences. In contrast to 5′ ends, the mapping of 3′ ends is arbitrary, because in most cases the transcription level is decreased significantly from 5′ to 3′ ends of cis-encoded ncRNAs. In other words, many putative antisense RNAs may represent series of transcripts with varying lengths; therefore, we manually assigned 3′ ends to the points where the transcription level was almost decreased to 0. The length of these transcripts varied significantly (the median, 281 nt), and some of them were localized to a single gene, as in the case of asMAV_0554 or asMAV_3303 (Figures 2B and 2C), whereas others spanned several adjacent genes, as in the case of asMAV_1569–1571 (Figure 2D). We checked all the detected asRNAs for ORFs longer than 70 nt. Potential polypeptides were subjected to blastp searches in the “Non-redundant protein sequences” database. The results showed that none of the detected asRNAs had ORFs that code for protein homologs in the database. While several transcripts showed relatively high transcription levels, as in the case of asMAV_3303, asMAV_1569–1571 spanning 3 genes showed much higher transcription levels than antisense genes. The full list of identified putative antisense RNAs is provided in Table S3.

#### Intergenic sRNAs

Visual inspection of the transcriptional profile allowed the identification of several trans-encoded sRNAs (Table 4). Each of them was transcribed at a much higher level than its neughboring genes and antisense RNA. Overall, their expression exceeded the expression of protein-coding genes (Table 1). The expression of each trans-encoded sRNA was further confirmed by quantitative qRT-PCR (Table S4). The correlation coefficient between RPKM values and qRT-PCR was acceptable (*r* = 0.75). Some discrepancies between RPKM values and qRT-PCR could be due to non-optimal PCR primers (short lengths of RNAs) and their tight secondary structures. The expression and lengths of igMAV_1415–1416, igMAV_2868–2869, and igMAV_1034–1035 were confirmed by northern blotting (Figure S1). In our earlier work, we used direct cloning and sequencing to reveal and map 4 abundant intergenic transcripts, igMAV_0380–0381, igMAV_1034–1035, igMAV_1415–1416, and igMAV_1531–1532 (processed 5′ UTR of the 16S r RNA gene). Subsequently, 5′ and 3′ RACE were used to precisely map these transcripts [23]. The combination of RACE in the earlier work and visual inspection of the transcriptional profile in the present study has allowed us to define the 5′ ends for these transcripts. The coordinates of 5′ ends mapped using these methods match almost precisely to each other; however, visual mapping identified slightly longer transcripts due to different points of 3′ ends. 3′ RACE is not as precise as 5′ RACE, and truncated transcripts have possibly been mapped in the earlier work.

**Table 4:** Trans-ncRNAs.

name	MTB homolog	start	end	type	RPKM
igMAV_0468-0469	MTS2823: *rv3661-rv3662c*	458799	458494	unknown	672182,73
igMAV_0469-0470	MTS2822 (B11, mpr19): *rv3660c-rv3661*	459933	460045	6C RNA	590414,35
igMAV_2215-2217	rnpB: *rv2226-rv2227*	2219895	2220299	rnpB	417173,33
igMAV_1415-1416*	MTS0997: *rv1264-rv1265*	1383757	1383620	unknown	357426,83
igMAV_0380-0381	C8: *tRNA-ser-rv3722c*	372509	372605	4.5S RNA	263130,70
igMAV1034-1035*	no homolog	973054	972924	unknown	142981,81
igMAV_2868-2869*	homolog not revealed (*rv1846c-1847*)	2895495	2895396	unknown	6035,19
igMAV_2936-2937	no homolog	2975038	2974831	unknown	1305,38
igMAV_4536-4537	no homolog	4663719	4663645	unknown	1011,07
igMAV_4914-4915	MTS0194: *rv0243-rv0244c*	5048801	5048678	unknown	458,49

For each trans-ncRNA MTB homolog and genes flanking it are shown if present. For igMAV_2868-2869 homolog in MTB has not been revealed, although the region coding for this transcript is conserved among these species. Coordinates of trans-ncRNA are presented according to MAH104 genome and their types are predicted according to RFAM database.

* Non-coding RNAs confirmed by Northern blotting.

### Intergenic sRNAs in *M. tuberculosis* and *M. avium*: common and different

**Figure 3:**
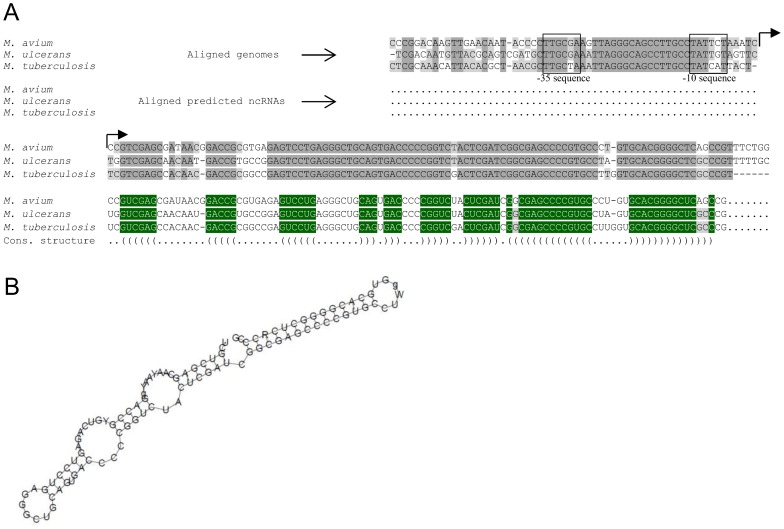
Intergenic sRNA igMAV_2868–2869 and its predicted homologs in *M. ulcerans* and *M. tuberculosis*. (A) Alignment of the intergenic region, MAV_2868–MAV_2869, coding for sRNA with its homologs in *M. ulcerans* (MUL_3032–3033) and *M. tuberculosis* (rv1846c–1847). Only regions coding for sRNA and their promoters are shown. Alignment of DNA sequences was performed with UGENE software [52]. The promoter regions containing –35 and –10 sequences and the coding regions are conserved among the 3 species. Alignment of sRNAs and their consensus secondary structures was performed with the WAR webserver [45] and is shown below the alignment. Nucleotides that are conserved among the 3 species and form duplex structures are highlighted in green. (B) Predicted consensus secondary structure of igMAV_2868–2869. Extensive complementary regions form the secondary structure with very low free energy.

The identified intergenic sRNAs of *M. avium* were subjected to a search for *M. tuberculosis* homologs. As a result, 6 of 10 sRNAs were found to have such homologs (Table 4). For igMAV_2868–2869, the sRNA is encoded by the intergenic locus that was flanked by genes conserved among *M. avium*, *M. tuberculosis* (rv1846–1847c), and other mycobacteria; however, surprisingly, this non-coding RNA has not yet been identified in *M. tuberculosis*. In this study, we aligned the sequences of this locus from *M. avium*, *M. tuberculosis*, and *M. ulcerans* using the WAR webserver [41] and found that a part of this locus, coding for the sRNA, is highly conserved among these organisms (Figure 3A). In addition to significant homology, extensive complementary regions were revealed, suggesting the importance of a secondary structure for this sRNA (Figure 3B). A possible reason for the lack of expression of this sRNA in *M. tuberculosis* is that the sRNA is regulated differently in *M. tuberculosis* in comparison with *M. avium* and therefore may be expressed under specific environmental conditions, which have not yet been used for the searching of sRNAs.

Six of the revealed intergenic sRNAs of *M. avium* were expressed at a much higher level than others. Among these, only igMAV_1034–1035 has no homologs in *M. tuberculosis*, suggesting that igMAV_1034–1035 regulates physiological aspects that are specific for *M. avium*, but not its pathogenic counterparts.

We searched for homologs of *M. tuberculosis* intergenic sRNAs in the *M. avium* genome by retrieving and aligning their sequences to the *M. avium* genome by BLAST with low-stringency parameters. To validate their presence or absence, we searched homologs of *M. tuberculosis* genes flanking these sRNAs in *M. avium* with the KEGG database. This method has been used to show that *M. avium* lacks several intergenic sRNAs found in *M. tuberculosis*, including MTS479, MTS1082, MTS1338, and MTS2975 [21]. Among them, MTS479 and MTS1338 are of special interest. MTS479 is one of the ncRNAs whose expression is increased in response to modeled oxidative stress, which mimics the intracellular environment. Transcription of MTS1338 is strongly induced in the stationary phase and is regulated by the DosRS two-component transcriptional regulator [20]. Genes regulated by DosRS constitute the so-called DosR regulon and are activated under conditions that do not allow aerobic respiration. These genes are assumed to play an important role in the adaptation of *M. tuberculosis* to host environments during infection. The lack of MTS479 and MTS1338 in conditionally pathogenic *M. avium* underlines their role in the pathogenesis.

### Expression of igMAV_0468–0469 and igMAV_0469–0470 in vivo

**Figure 4:**
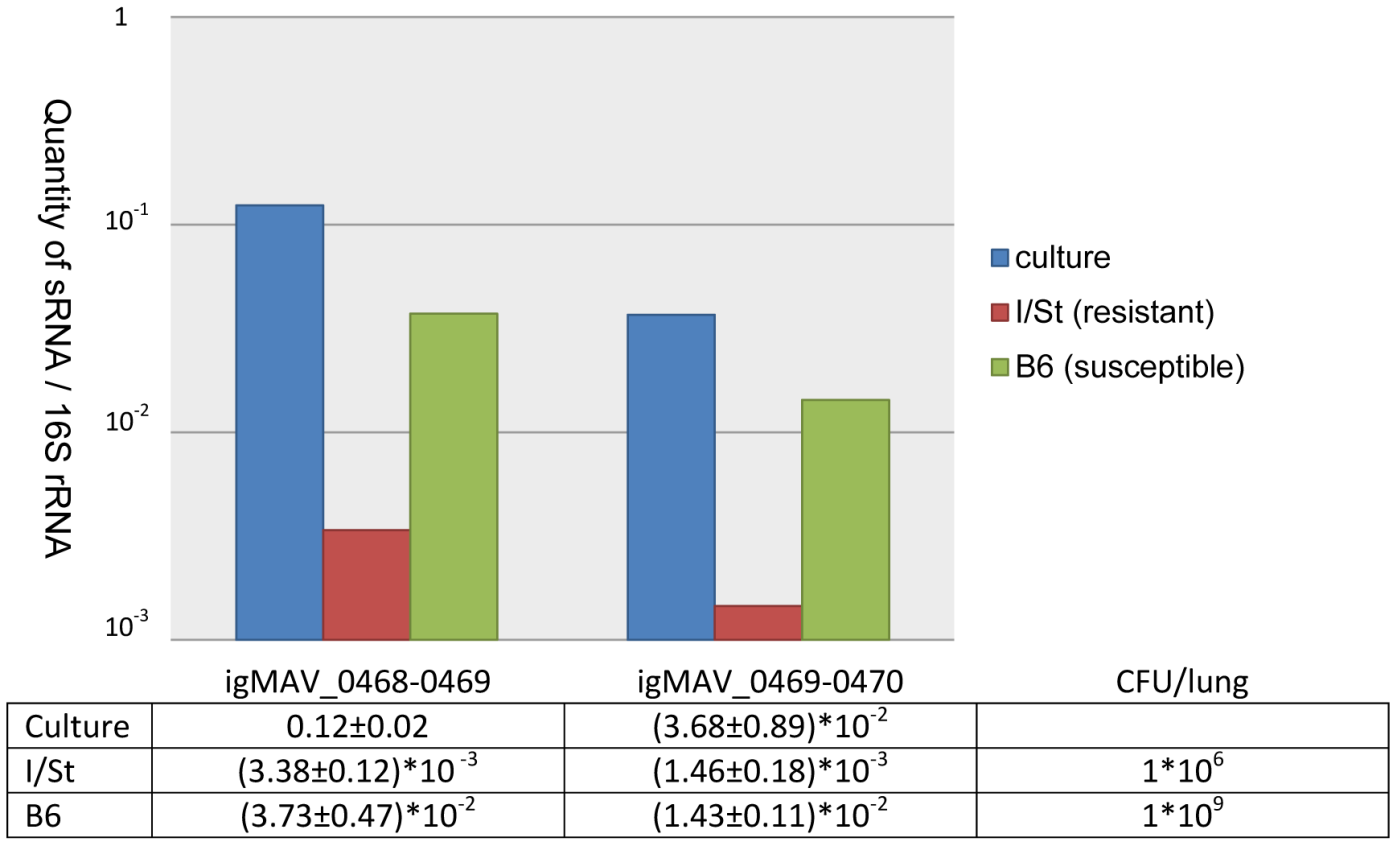
Expression of igMAV_0468–0469 and igMAV_0469–0470 in *M. avium* isolated from cultures or the lungs of resistant and susceptible mouse strains. The quantities of intergenic sRNAs relative to 16S rRNA are shown on a logarithmic scale. The relative quantities of intergenic sRNAs, as well as their confidence intervals, are summarized in the table below the figure. The CFU/lung values for I/St and B6 mice are also shown.

We selected 2 intergenic sRNAs with the highest expression level *in vitro*, igMAV_0468–0469 and igMAV_0469–0470, to assess by qRT-PCR their expression in the lung tissue of infected mice which previously were shown to be genetically susceptible and resistant to infection. Compared to I/St, B6 mice are considerably more susceptible to infection caused by *M. avium* in terms of bacterial multiplication and damage of the lung tissue: in B6, but not I/St mice prolonged infiltration with neutrophils leads to formation of necrotic lung granulomata and death [42]. The expression of igMAV_0468–0469 and igMAV_0469–0470 was very high in culture medium, only slightly lower in the lungs of susceptible, but dramatically reduced in the lungs of resistant mice (Figure 4). Interestingly, Arnvig et al. demonstrated that the transcript of MTS2823, the *M. tuberculosis* homolog of igMAV_0468–0469, accumulated at a high level comparable to that for 16S rRNA in the lungs of B6 mice which are relatively resistant to TB [20]. The different expression profiles of MTS2823 and ncMAV_0468–0469 in resistant mice may be explained either by differences in physiology between *M. avium* and *M. tuberculosis* or by differences in infection models. Regulation of MTS2823 expression has not been studied so far. It doesn’t contain known promoter sequences upstream of 5′ end [20], and further work on elucidation of regulatory mechanisms is needed. Although there are similarities in the infection models of *M. tuberculosis* (resistant B6 mice) and *M. avium* (resistant I/St mice), the 2 models are not identical. They differ in the regulation of the neutrophil response, which may be explained by different patterns of genetic control between 2 infections [43]. Specifically, the susceptibility of mice to *M. avium* infection depends on a single gene, *Nramp1*, which encodes a proton-coupled divalent metal ion transporter associated with bacterial phagosomes in macrophages [44]. In contrast, the susceptibility of mice to *M. tuberculosis* infection is always controlled by many genetic loci [45]. These differences may lead to different microenvironments inside host macrophages, thereby causing different transcriptional responses.

## Conclusion

In summary, we performed massive sequencing on the Illumina platform to obtain a comprehensive description of *M. avium* transcriptome. The qualitative and quantitative analysis reveals the abundance of non-coding RNAs, which even exceeds the amount of transcribed coding sequences. We found that 6 *M. avium* intergenic sRNAs were expressed at extremely high levels, compared to other sRNAs and the majority of protein-coding genes. Among them, the expression of *igMAV_0468–0469* and *igMAV_0469–0470* depends on the host genetics, which was demonstrated in *in vivo* experiments. These results may imply the involvement of these intergenic sRNAs in the adaptation of mycobacteria to the host defense system.

In recent years, many bacterial sRNAs have been shown to control the overall response to stress and act as substantial regulators of bacterial virulence [46], [47]. sRNAs MTS479 and MTS1338, which are implicated in pathogenesis, are present in pathogenic *M. tuberculosis*, but not in conditionally pathogenic *M. avium*. Their expression may have an impact on pathogenesis.

The new era of sequencing technologies in the near future will provide researchers with a useful tool for acquiring transcriptional maps of many, if not all, bacteria. A systematic comparison of their expression profiles at different stages of infection will help assess the influence of sRNAs on different virulence phenotypes.

## Materials and Methods

### Ethics statement

Mice of inbred strains I/StSnEgYCit (I/St) and C57BL/6YCit (B6) were bred and maintained under conventional, non-specific-pathogen-free conditions at the Animal Facilities of the Central Institute for Tuberculosis (Moscow, Russia) in accordance with guidelines from the Russian Ministry of Health (guideline 755) and the NIH Office of Laboratory Animal Welfare (assurance A5502-06). Water and food were provided *ad libitum*. Female mice aged 2.5–3.0 mo. at the beginning of experiments were used. All experimental procedures were approved by the Central Institute for Tuberculosis Institutional Animal Care Committee (IACUC), protocols 6, 7, 8 11, 14 of March 6, 2012.

### Strains and culture conditions

A previously characterized [48] chicken isolate *M. avium* subsp. *avium* strain TMC724 (ATCC25291) was a kind gift of T. Ulrichs, Max Planck Institute for Infectious Biology, Berlin, Germany. Bacilli were grown at 37°C in Dubos broth with addition of 0.05% Tween 20 for approximately 2 weeks until logarithmic growth phase (OD_600_ ∼ 0.6) was achieved.

### Infection and quantitative real-time PCR

Two groups of 5 female I/St and B6 mice were infected with 1–2×10^3^ viable CFU of *M. avium* in the aerosol chamber as described [42]. 13 weeks following challenge, lungs were homogenized in Trizol reagent (Life Technologies, Carlsbad, CA) and RNA was isolated according to a standard protocol using BeadBeater cell disrupter with 0.1 mm zirconia/silica beads (BioSpec Products, Bartlesville, OK). cDNA for qRT-PCR was made with random primers and Superscript II according to manufacturer’s instructions (Life Technologies). qRT-PCR was performed using Stratagene MX3005p machine (Agilent Technologies, Santa Clara, CA) and qPCRmix-HS SYBR reagent (Evrogen, Moscow, Russia). PCR primers are listed in Table S5. All amplifications were repeated in triplicates.

### RNA isolation and mRNA enrichment

Three independently grown bacterial cultures were cooled rapidly on ice and centrifuged. RNA was isolated as previously described [49]. Briefly, cell pellets were resuspended in Trizol reagent (Invitrogen) and shaked 3 times for 30 sec. at BeadBeater cell disrupter with 0.1 mm zirconia/silica beads. RNA was isolated using phenol-chloroform extraction and re-suspended in mQ water. RNA isolated from 3 cultures was mixed and treated with Turbo DNase (Life Technologies) to remove traces of genomic DNA and purified with the RNeasy mini kit (Qiagen, Venlo, Netherlands). The absence of DNA contamination was verified by PCR with primers specific to 16S rRNA. RNA sample was depleted of 16S and 23S rRNA with MicrobExpress kit (Life Technologies) and ethanol precipitated. Depletion of 16S and 23S rRNA was verified by the agarose gel electrophoresis.

### RNA processing and illumina sequencing

To prepare cDNA for sequencing we applied “Illumina RNA ligation protocol” described in [24] with slight modifications. Briefly, 200 ng of RNA was fragmented with RNA fragmentation reagent (Life Technologies) by heating at 70°C for 10 min. After ethanol precipitation, RNA was treated with Shrimp alkaline phosphatase (Fermentas, Vilnius, Lithuania) to remove 5′ and 3′ phosphate groups including threephosphate groups from the 5′ ends of RNA molecules. RNA was purified with RNeasy mini kit (Qiagen) according to modified protocol from “Directional mRNA-Seq Sample Preparation guide” issued by Illumina. RNA was 5′- phosphorylated using T4 Polynucleotide kinase (Fermentas), ethanol precipitated and dissolved in mQ water. 3′- and 5′- RNA adapters were ligated and cDNA was constructed using TruSeq™ Small RNA Sample Preparation kit (Illumina, San Diego, CA). After 12 cycles of PCR amplification, cDNA constructs were fractionated in polyacrylamide gel. 65–75-nt-long cDNA fragments were excised, eluted from the gel and sequenced by running 76 cycles on the Illumina Genome Analyzer IIx.

### Processing of RNA-seq data

Before mapping of reads to the genome, terminal sequences of adapters were trimmed with Perl script. The reads were mapped to M. avium subsp. hominissuis 104 genome (GenBank accession number cp000479.1) with Bowtie 2 [50], setting parameters: *-q –sensitive-local*. Mapping statistics and RPKM values [33] for annotated genes and trans-encoded ncRNAs was calculated using custom Perl scripts. Reads overlapping a gene at least by one nucleotide were accounted for when calculating RPKM for this gene. Transcriptional profiles for forward and reverse strains of genome were generated, representing count of overlapping reads for every nucleotide of the genome. The transcriptional profile was visualized with Artemis genome browser [26].

To search for genomic loci, present *M. avium hominissuis 104* but absent in *M. avium subsp. avium* TMC724, we aligned genomes of these strains with Nucmer package, a part of MUMmer 3.0 software [51]. Textual output was parsed with Perl script to retrieve coordinates of these loci. They were compared with non-transcribed regions of genome to reveal MAH-specific LSPs.

Abrupt increases of transcription level were considered to be putative TSPs. They were mapped with Perl script, which considered an increase to be a putative TSP, if it met several empirical criteria: (i) more than twofold rise of transcription in 3-nucleotide window upstream the increase (ii) transcription level at the highest point of the increase is higher than at every point in 60 nt window upstream of the increase (iii) transcriptional level at the highest point of the increase is more than 10. All found putative TSPs were manually verified. TSPs located in intergenic loci were considered to be TSPs of their respective genes. TSPs located in antisense orientation to the genes were considered to be TSPs of cis-encoded ncRNAs.

### Accession codes

Sequence read data, transcriptional profile for visualization in Artemis genome browser and RPKM values for annotated genes have been submitted to the GEO database at NCBI under accession number GSE46281.

### 5′ RACE and northern blotting

For verification of the revealed TSPs five genes were randomly selected (MAV_1369, MAV_3090, MAV_4124, MAV_0525, MAV_1669) and 5′ RACE was made using FirstChoice® RLM-RACE Kit (Life Technologies). Products of 5′RACE were amplified with external and internal primers (Table S5) and sequenced.

Northern blotting for confirmation of presence of trans-encoded ncRNAs MAV_1415-1416, MAV_2868-2869 and MAV_1034-1035 was made using riboprobes generated with Riboprobe® Systems (Promega, Fitchburg, WI). Primers for generation of DNA templates for riboprobe generation are listed in Table S5.

## Supporting Information

Figure S1 **Northern blots of intergenic sRNAs igMAV_1034-1035, igMAV_1415-1416 and igMAV_2868-2869.**
(PDF)

Table S1 **RPKM values.** RPKM values for sense and antisense transcription are given for MAH104 genes present in MAA TMC724.
(XLSX)

Table S2 **Transcription start sites.** TSS are given for MAH104 genes present in MAA TMC724. -10 consensus sequence if present, coordinate of TSS and the length of 5′-UTR are given for each gene.
(XLSX)

Table S3 **Cis-ncRNAs.** The coordinates of 5′ and 3′ ends, genes antisense to ncRNAs and products of these genes are given for each cis-ncRNA.
(XLSX)

Table S4 **Results of qRT-PCR on intergenic sRNAs.** The quantity of each sRNA is given according to qRT-PCR (relative to 16S rRNA) and RNA-seq (RPKM value). The correlation coefficient between RPKM values and qRT-PCR results is also shown.
(XLSX)

Table S5 **Oligonucleotides.**
(XLSX)
