# Supplementary material for: RNA-Seq Analysis of Mycobacterium avium Non-Coding Transcriptome
Source: PLoS One. 2013 Sep 16;8(9):e74209. doi: 10.1371/journal.pone.0074209 (PMC3774663; doi:10.1371/journal.pone.0074209)
Supplement: Figure S1 — Northern blots of intergenic sRNAs igMAV_1034-1035, igMAV_1415-1416 and igMAV_2868-2869. (PDF) [file pone.0074209.s001.pdf]

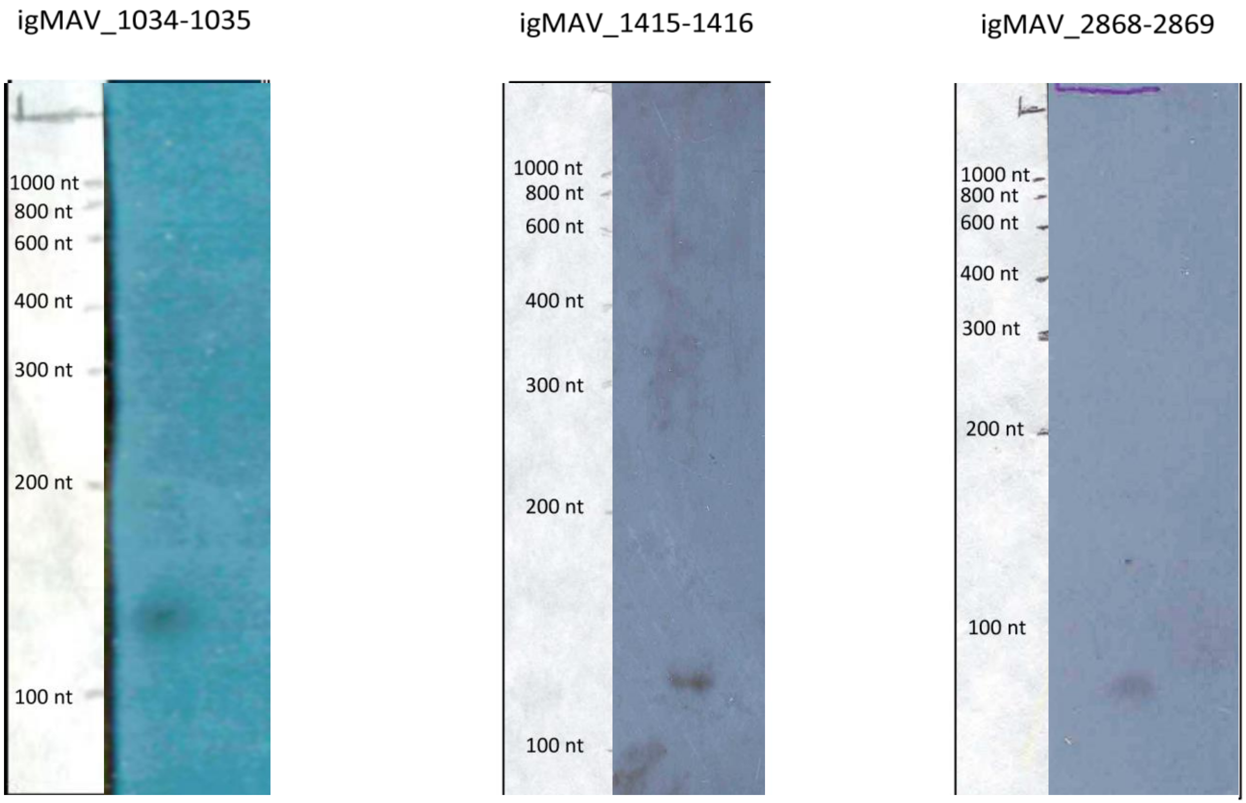

Suppl. figure: Northern blots of intergenic small RNAs igMAV_1034-1035, igMAV_1415-1416 and igMAV_2868-2869. Sizes of the bands of RiboRuler Low Range RNA Ladder (Thermoscientific) are shown.
